# Integrating deep learning features from mammography with SHAP values for a machine learning model predicting over 5-year recurrence of breast ductal carcinoma *In Situ* post-lumpectomy

**DOI:** 10.3389/fimmu.2025.1681072

**Published:** 2025-09-15

**Authors:** Yupeng Sha, Quan Yuan, Yi Du, Shuqi Yang, Ming Niu, Xiaoshuan Liang, Shanshan Sun, Tong Li, Shu Gong, Jiguang Han

**Affiliations:** ^1^ Department of Breast Surgery, Harbin Medical University Cancer Hospital, Harbin, Heilongjiang, China; ^2^ Quanzhou First Hospital Affiliated to Fujian Medical University, Quanzhou, Fujian, China

**Keywords:** ductal carcinoma *in situ*, breast-conserving surgery, mammography, deep learning, recurrence

## Abstract

**Background:**

In women with ductal carcinoma *in situ* (DCIS) undergoing breast-conserving surgery, still part will progress to invasive breast cancer (IBC) in the future. Mammograms offer rich tumor data for patient stratification, but current prediction methods focus on clinicopathological factors, overlooking imaging insights.

**Methods:**

We retrospectively analyzed 140 DCIS patients from Harbin Medical University Cancer Hospital (2011-2020, followed up to 2025). Preoperative digital mammograms and clinicopathological data were collected, with mammographic features extracted using pyradiomics and supervised by a senior radiologist. Feature selection employed 10-fold cross-validated LASSO regression. The dataset was split into training (n=100) and validation (n=40) sets (10:4 ratio). Sixteen machine learning algorithms combining mammographic deep learning features and clinicopathological variables were developed and compared for predicting DCIS recurrence. Model performance was assessed using ROC, sensitivity, specificity, PPV, NPV, and SHAP values for interpretation.

**Results:**

The Gradient Boosting Machine (GBM) algorithm had the best predictive performance, with an AUC of 0.918 (95% CI 0.873-0.963) in the test set. SHAP values indicated that the mammographic signature (MS) was the most significant predictor, followed by Ki-67 index and histological grade. Patients not receiving radiotherapy had higher recurrence rates than those who did. Decision curve analysis validated the model’s clinical utility across various risk thresholds.

**Conclusion:**

Our study developed an interpretable GBM model incorporating mammographic and clinical data to predict DCIS recurrence (AUC = 0.918). Key predictors were mammographic signature, Ki-67, and tumor grade, offering clinicians a practical tool for personalized postoperative management.

## Introduction

Breast cancer (BC) represents roughly one-third of all female malignant neoplasms globally (1). The progressive advancement of diagnostic technologies has led to enhanced detection rates of breast ductal carcinoma *in situ* (DCIS), which currently constitutes 20%–25% of all recently identified BC diagnoses (2). Breast-conserving surgery (BCS) has become the main local treatment for DCIS to achieve precise excision with the smallest possible margin and to minimize trauma (3). Despite the historically positive outlook for DCIS cases, comprehensive large-cohort analyses have demonstrated the risk of ipsilateral recurrence subsequent to breast-conserving treatment in individuals diagnosed with DCIS (4). Consequently, precise identification of DCIS patients who face elevated recurrence risk following breast-conserving surgery represents a critical factor in establishing appropriate postoperative therapeutic strategies. Mammography is the cornerstone of DCIS screening and diagnosis. The core functions include early detection, risk stratification, and treatment guidance (5). Mammography is highly sensitive to calcified DCIS, and > 90% of female patients with DCIS, especially high-grade DCIS, show suspicious microcalcifications on mammograms (6, 7). Consequently, the systematic collection and examination of mammographic characteristics, including lesion dimensions and Breast Imaging Reporting and Data System (BI-RADS) categorization, represents a critical component in the precise assessment of DCIS recurrence probability. Recent researches has established that integrating deep learning (DL) methodologies with radiological data represents a novel diagnostic and therapeutic strategy for BC addressing the under exploitation of visual diagnostic information (8). Khalid proposed an efficient DL model to recognize BC in computerized mammograms of varying densities (9). Alaeikhanehshir et al. used DL in mammography to distinguish between high- and low-risk DCIS, enabling active surveillance of patients (10). Nevertheless, contemporary DL investigations predominantly emphasize multimodal feature representation extraction, while the intricate architecture of opaque computational models impedes comprehensive understanding of individual feature contributions to predictive outcomes. The lack of direct and effective explanations limits their impact on clinical decision-making.

Shapley additive explanations (SHAP) value interpretation is a new function-based interpretability method that provides a deeper understanding of the key predictors of machine learning (ML) models, thereby improving their transparency and credibility (11). In the present study, we reviewed 140 the data of patients with DCIS who underwent BCS at a large cancer center and integrated the extracted DL features from mammography and other clinicopathological features to construct an ML model for predicting DCIS recurrence > 5 years after lumpectomy. Finally, by combining the SHAP values, we visually explained the potential factors affecting the long-term recurrence of DCIS.

## Patients and methods

### Study population

The research received ethical clearance from the Institutional Review Board at Harbin Medical University Cancer Hospital (reference: YD2024-18) and was conducted in full compliance with the ethical standards established by the Declaration of Helsinki. Because this was a retrospective study and all data were anonymized, patient informed consent was waived.

This retrospective investigation incorporated 140 female patients with confirmed primary DCIS who received inpatient treatment at the Affiliated Cancer Hospital of Harbin Medical University during the period spanning March 1, 2011, through March 1, 2020.All patients had complete pathological and laboratory test results and clear mammography images. Data including mammographic features, patient characteristics, laboratory results, pathological results (according to the American Joint Committee on Cancer (AJCC) Cancer Staging Manual, 8th edition), and treatment strategies were collected (**Table 1**).

**Table 1:** Performance of multiple machine learning models for identifying breast cancer recurrence status based on molybdenum target features.

Model_name	AUC	95% CI	Sensitivity	Specificity	Accuracy	PPV	NPV	Task
LR	0.829	0.738-0.921	0.917	0.617	0.702	0.489	0.949	train
	0.622	0.417-0.826	0.600	0.711	0.679	0.300	0.889	test
NaiveBayes	0.826	0.733-0.920	0.792	0.700	0.726	0.514	0.894	train
	0.704	0.523-0.885	0.500	0.911	0.821	0.500	0.891	test
SVM	0.868	0.784-0.951	0.958	0.746	0.798	0.590	0.978	train
	0.678	0.485-0.872	0.600	0.761	0.732	0.353	0.897	test
KNN	0.836	0.752-0.921	0.583	0.914	0.798	0.667	0.841	train
	0.638	0.456-0.820	0.900	0.341	0.429	0.225	0.937	test
DecisionTree	1.000	1.000-1.000	1.000	1.000	1.000	1.000	1.000	train
	0.702	0.532-0.872	0.600	1.000	0.768	0.400	0.902	test
RandomForest	0.891	0.819-0.963	0.750	0.933	0.881	0.818	0.903	train
	0.630	0.430-0.831	0.600	0.800	0.750	0.375	0.900	test
ExtraTrees	1.000	1.000-1.000	1.000	1.000	1.000	1.000	1.000	train
	0.687	0.498-0.876	0.500	0.844	0.768	0.385	0.884	test
XGBoost	0.992	0.980-1.000	1.000	0.933	0.952	0.857	1.000	train
	0.711	0.500-0.922	0.700	0.761	0.750	0.389	0.921	test
LightGBM	0.877	0.805-0.949	0.875	0.857	0.821	0.636	0.941	train
	0.634	0.424-0.843	0.600	0.833	0.732	0.353	0.897	test
GradientBoosting	0.975	0.949-1.000	0.958	0.933	0.940	0.852	0.982	train
	0.704	0.507-0.902	0.600	0.867	0.804	0.462	0.907	test
AdaBoost	0.963	0.929-0.997	0.875	0.917	0.905	0.808	0.948	train
	0.730	0.540-0.921	0.600	0.867	0.804	0.462	0.907	test
MLP	0.844	0.759-0.930	0.958	0.583	0.690	0.479	0.972	train
	0.650	0.436-0.865	0.600	0.778	0.732	0.353	0.897	test

Inclusion criteria comprised: (1) pathologically confirmed DCIS who underwent BCS; (2) high-quality digital mammography images before treatment; (3) comprehensive clinical information (including chemotherapy regimens, radiation treatment protocols, hormonal therapeutic interventions, hormone receptor and human epidermal growth factor receptor 2 [HR/HER2] expression profiles, Ki-67 proliferation index, and histopathological grading); (4) complete pathology information;(5) All patients were pathologically confirmed to have negative margins after tumor resection.

Exclusion criteria comprised: (1) distant metastasis or invasive carcinoma; (2) other malignant tumors; (3) missing key data (e.g., imaging or molecular markers); (4) history of breast radiotherapy or loss to follow-up.

The follow-up endpoint of this study was April 1, 2025. Following the completion of appropriate therapeutic interventions, patients underwent systematic monitoring through clinical consultations, telephonic communication, or electronic correspondence at three-month intervals during the initial six-month period, subsequently at six-month intervals for a maximum duration of five years, and thereafter on an annual basis, with the principal objective of identifying disease recurrence.

### Imaging acquisition and interpretation

Digital mammographic imaging was performed utilizing Mammomat Novation DR (Siemens AG Medical Solutions, Erlangen, Germany) and Selenia Dimensions (Hologic, Bedford, Mass, USA) systems, incorporating both craniocaudal (CC) and mediolateral oblique projections. The region of interest (ROI) showing the most suspicious lesion in the CC-view for each patient was prioritized. To ensure reliable and reproducible BI-RADS categorization, two experienced radiologists conducted independent evaluations of all imaging studies. (R4-R6 with ≥ 8 years of mammography experience, respectively). Consensus regarding the final diagnostic assessment was achieved through collaborative discussion when interpretive differences arose. The interpreting radiologists remained unaware of histopathological findings while retaining access to relevant clinical data and previous imaging studies. Based on the 2013 American College of Radiology Breast Imaging Reporting and Data System (BI-RADS) classification framework, lesions designated as categories 2 or 3 were characterized as benign or likely benign entities, while those assigned categories 4 or 5 were classified as potentially malignant findings warranting histopathological confirmation.

### Data preprocessing

Calcified regions in the mammography images were annotated as follows: for diffusely distributed calcifications, the entire area was uniformly annotated; for multiple independent calcification clusters, the specific cluster indicated for biopsy in the radiology report was prioritized; and for large calcified areas, the entire scope was annotated. All calcification region annotations were independently completed by two trained annotators (SA: MD; MM: medical technology researcher) using 3D Slicer software (version 4.10.2) on full images supervised by a senior breast radiologist (RM).

Tumor recurrence encompassed both localized recurrence and metastatic spread to distant tissues or organs. Localized recurrence was characterized as tumor reappearance within the ipsilateral breast, chest wall, or corresponding regional lymph nodes. Neoplasm classification was conducted in accordance with the eighth edition of the AJCC staging criteria. All lymph node-positive (LMN+) cases were confirmed pathologically. Based on the established criteria from the American Society of Clinical Oncology (ASCO) and the College of American Pathologists (CAP), estrogen receptor and progesterone receptor positivity were characterized as ≥ 1% of tumor cell nuclei demonstrating positive staining. HER2 expression was determined in accordance with the 2018 ASCO/CAP criteria, whereby immunohistochemistry scores of 3+ are classified as positive, while scores of 2+ are deemed positive when HER2 gene amplification is confirmed through fluorescence *in situ* hybridization (FISH) analysis. Four serum inflammation- and immunity-related biomarkers were measured: the ratio of platelets to lymphocytes (PLR), the ratio of neutrophils to lymphocytes (NLR), the ratio of lymphocytes to monocytes (LMR), and the platelet-albumin ratio (PAR, calculated as the quotient of platelet count and serum albumin concentration). All blood cell counts were performed using automated hematology analyzers (Sysmex XN series or Beckman Coulter DxH), and serum albumin was measured using standardized biochemical analysis methods (e.g., bromocresol green method or immunoturbidimetry), following strict clinical laboratory standard operating procedures.

### Machine learning model development

First, considering the different measurement units among variables, all variables were normalized using “StandardScaler.” Subsequently, to address feature dependency, Spearman’s correlation analysis was performed. When the correlation coefficient between any two variables exceeded 0.9, one variable was removed from the analysis. Between-group comparisons were conducted using the Mann–Whitney U test. Categorical data are presented as percentages (%), with Pearson’s chi-square analysis employed to assess between-group variations. The sample dataset was partitioned into training (n = 100) and internal validation (n = 40) cohorts using a 10:4 allocation ratio (12). Owing to the high-dimensional nature of features that adversely affect DCIS recurrence prediction, we sought to identify the features most closely associated with DCIS recurrence in the training set. Feature extraction was performed utilizing the “pyradiomics” module within Python 3.8.1, while feature selection was conducted through the least absolute shrinkage and selection operator (LASSO) algorithm. The optimal lambda parameter for feature selection was established via 10-fold cross-validation methodology (13). The prediction models were developed employing sixteen machine learning algorithms: partial least squares (PLS) (14), random forest (RF) (15), decision tree system (DTS) (16), support vector machine (SVM) (16), logistic regression (LR) (17), K-nearest neighbors (KNN) (18), eXtreme gradient boosting (XGBoost) (19), gradient boosting machine (GBM) (20), neural network (NeuralNet) (21), generalized linear model boosting (glmBoost) (22), naïve Bayes (23), decision tree (16), extra trees (24), light gradient boosting machine (25), adaptive boosting (AdaBoost) (26), and multilayer perceptron (27). To maintain model reliability across both training and testing datasets, a ten-fold cross-validation approach was implemented (13). To identify the optimal hyperparameters for each algorithm, a systematic grid search methodology was employed, utilizing the maximum area under the receiver operating characteristic (ROC) curve (AUC) (28, 29) as the evaluation metric for determining the superior model configuration. The Delong test was employed for AUC comparisons. The optimal model was constructed using the training dataset and subsequently evaluated through both internal and external validation datasets. Model efficacy was assessed on both training and testing datasets through the utilization of receiver operating characteristic curves, along with measurements of sensitivity, specificity, positive predictive value, and negative predictive value (28). To mitigate overfitting and enhance model generalization, rigorous regularization techniques were implemented during training. These included penalty-based complexity constraints, feature coefficient compression, and built-in regularization methods such as tree depth limits and randomized subspace sampling. All feature selection and hyperparameter tuning were conducted internally within the training set using repeated cross-validation to prevent data leakage. The final model performance was evaluated on a strictly retained validation set. Despite moderate sample sizes relative to initial feature dimensions, this study ensured an optimal event-to-prediction ratio through substantial dimensionality reduction and regularization. Further external validation in larger cohorts is required to confirm the model’s robustness and generalization capabilities. Additionally, decision curve analysis (DCA) was conducted to evaluate genuine clinical applicability. SHAP analysis was employed to elucidate the individual feature contributions to predictive outcomes (28, 29). The SHAP values obtained for representative cases demonstrated how specific features influenced particular samples, thereby facilitating comprehension of the model’s decision-making mechanisms (29). Subsequently, recursive feature elimination (RFE) was implemented to conduct additional variable selection and construct a streamlined model variant.

### Statistical analysis

Statistical analyses were conducted using R Studio version 4.3.3 and Jupyter Notebook 5.6.0. For categorical data, chi-squared or Fisher’s exact tests were used (30).Continuous variables were tested for normality using Shapiro-Wilk tests(a= 0.05). For normally distributed data, independent t-tests were used; otherwise, Mann-Whitney U tests were applied. Statistical significance was defined as p < 0.05.

## Results

**Figure 1:**
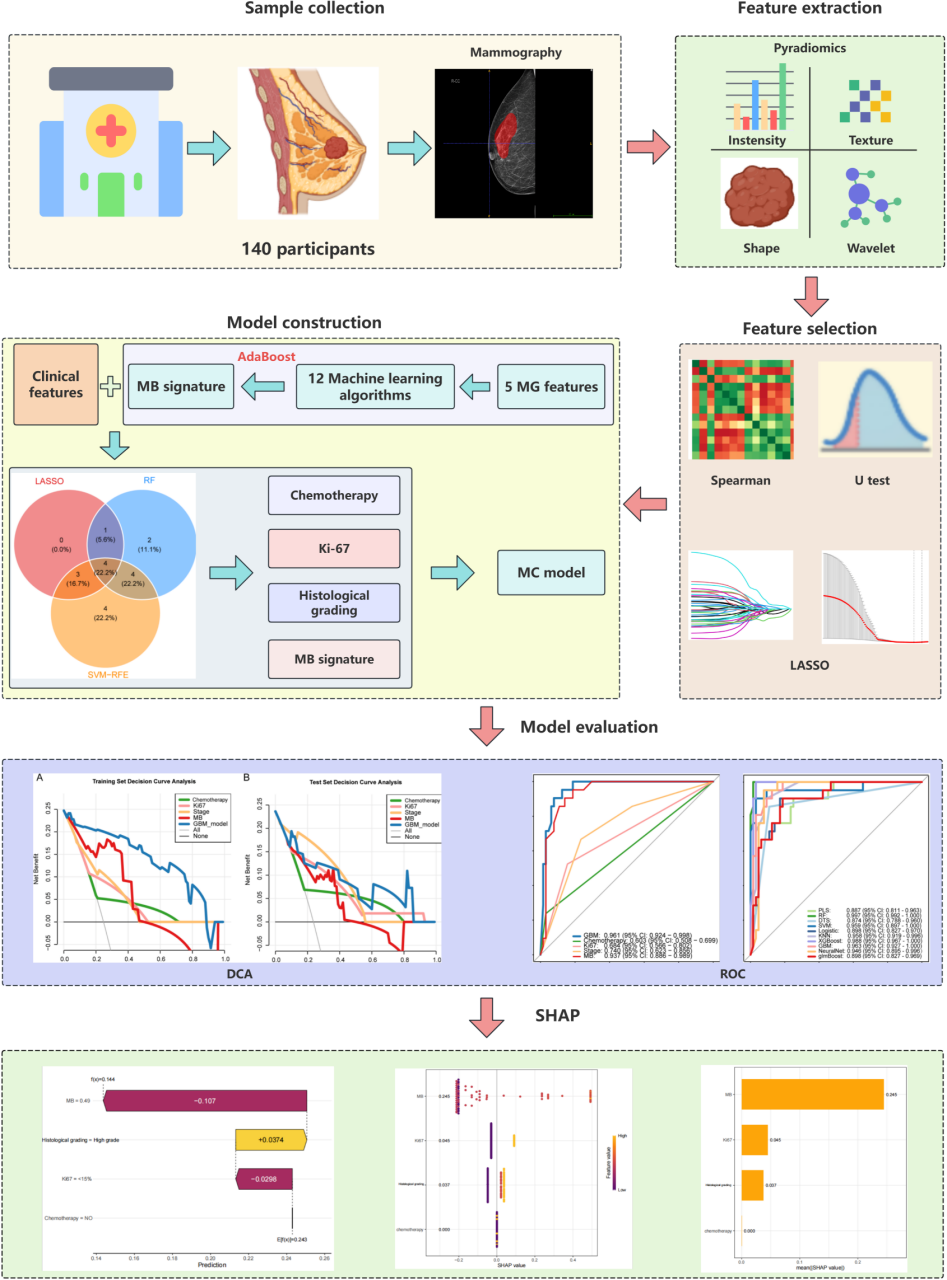
Brief technical flowchart.

The research methodology is illustrated in **Figure 1**.

### Baseline characteristics

Between March 2011 and March 2020, 140 patients with DCIS confirmed by biopsy or postoperative pathology were included. **Supplementary Table S1** presents the demographic and clinical characteristics of the study population at baseline. The most common histological grades were low (43.6%), high (35.7%), and intermediate (20.7%). Compared with patients with non-recurrence, patients with recurrence showed significantly higher histological grades and Ki67 indices; additionally, a higher proportion of these patients did not receive chemotherapy (**Supplementary Table S1**). All clinical characteristics (21 clinical features) were obtained from the electronic medical records of patients with DCIS.

### Feature selection

**Figure 2:**
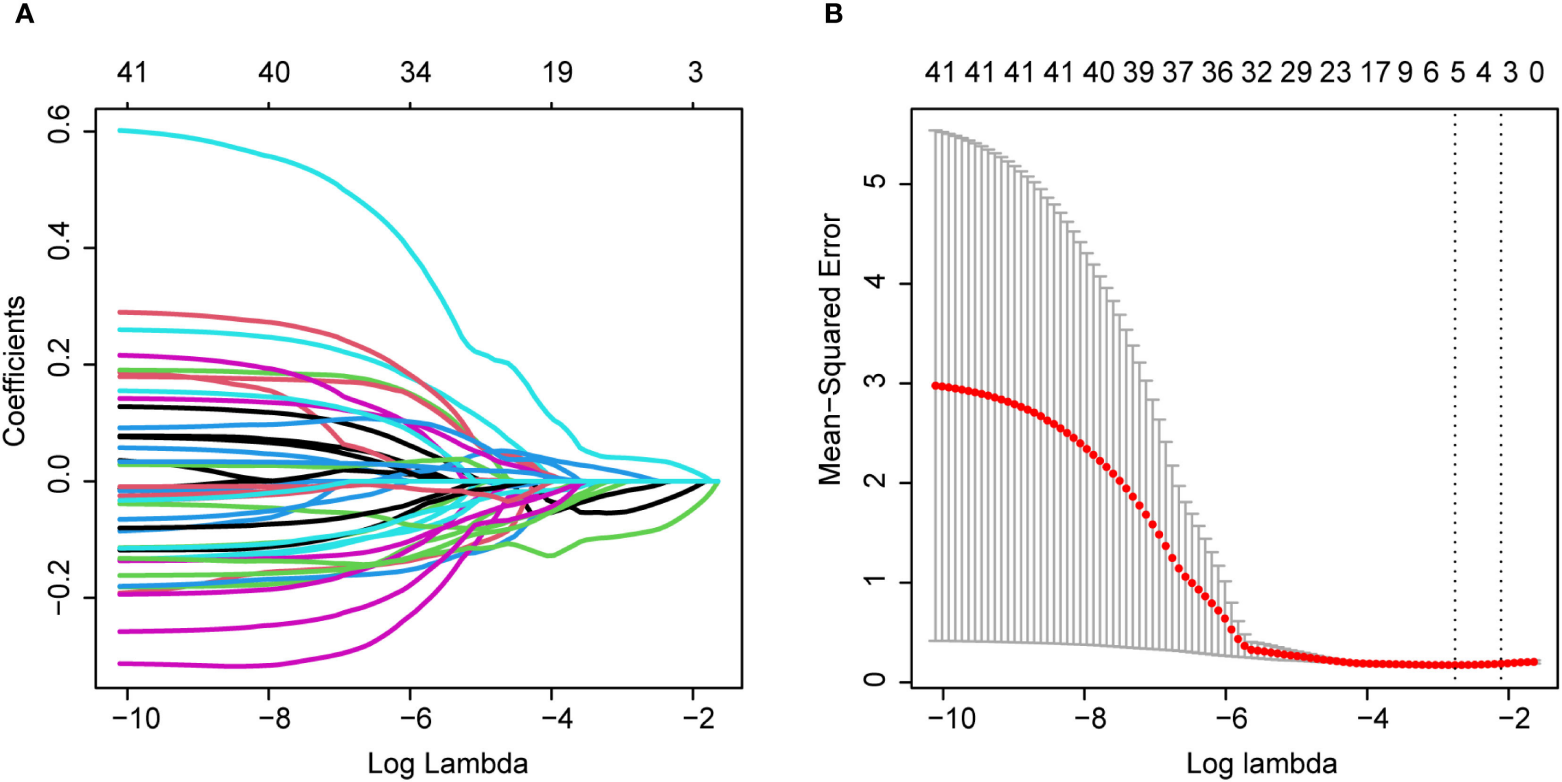
**(A)** and **(B)** LASSO coefficient convergence paths.

We performed radiomic feature extraction using the pyradiomics module in Python 3.8.1, obtaining a total of 849 features including shape features, first-order histogram features, and second-order texture features (**Supplementary Table S2**). To address the potential adverse effects of high-dimensional data on predicting breast intraductal carcinoma recurrence, we first standardized all features using StandardScaler to eliminate measurement unit discrepancies. Subsequently, we conducted Spearman correlation analysis (threshold ρ>0.9) to remove redundant features, resulting in 172 optimized features (**Supplementary Table S3**). Further refinement was achieved through Mann-Whitney U tests (p<0.05), which identified 41 statistically significant features (**Supplementary Table S4**). After partitioning the dataset into training and validation sets at a 10:4 ratio (**Supplementary Tables S5**, **S6**), we employed LASSO regression (**Figure 2A**) with 10-fold cross-validation (**Figure 2B**) to ultimately determine five optimal mammography (MG) features. The five selected radiomic features were: original firstorder 10Percentile (10th percentile from the first-order statistics of the original image), original glcm Contrast (contrast from the gray-level co-occurrence matrix), original glcm Idmn (inverse difference moment normalized from the gray-level co-occurrence matrix), wavelet HLL firstorder Median (median from the first-order statistics of the wavelet High-Low-Low filtered image), and wavelet HHL firstorder Median (median from the first-order statistics of the wavelet High-High-Low filtered image).

**Figure 3:**
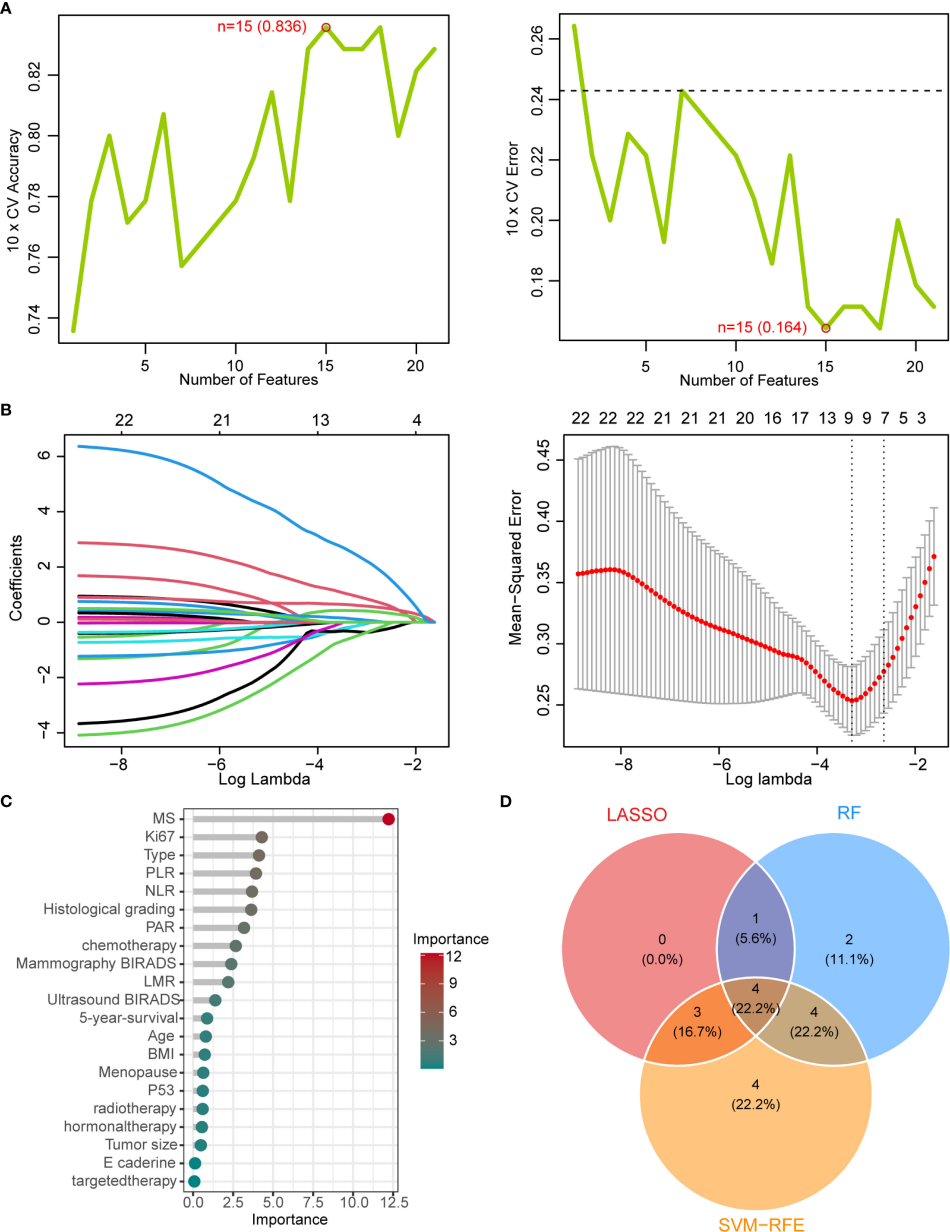
Machine learning-based feature selection **(A)** SVM-RFE algorithm performance showing accuracy (83.6%) and error rate (16.4%) with top 15 selected features. **(B)** LASSO coefficient convergence paths. **(C)** Random Forest feature importance ranking (threshold >1). **(D)** Venn diagram of overlapping features from LASSO, RF, and SVM-RFE methods, identifying four key predictors: chemotherapy status, Ki-67 index, histological grade, and mammographic signature (MS).

Based on these features, we evaluated twelve machine learning algorithms (**Table 1**), among which the AdaBoost algorithm demonstrated superior performance and was selected to establish the mammography signature (MS). To enhance the robustness of our predictive model, we further integrated MS with clinical features and blood inflammatory markers (PLR, NLR, LMR, and PAR). Feature selection was performed using three distinct methods: SVM-RFE, LASSO, and random forest. The SVM-RFE approach identified 15 optimal variables (accuracy: 83.6%; **Figure 3A**, **Supplementary Table S7**), LASSO selected 8 key variables (**Figure 3B**, **Supplementary Table S8**), and random forest determined 11 important variables (**Figure 3C**). Comprehensive analysis ultimately identified four core predictive variables: chemotherapy status, Ki-67 index, histological grade, and MS (**Figure 3D**), which served as the foundational elements for constructing our predictive model. This systematic feature selection and model development process ensured methodological rigor while significantly improving the reliability of predictive outcomes.

### Model performance comparison

**Figure 4:**
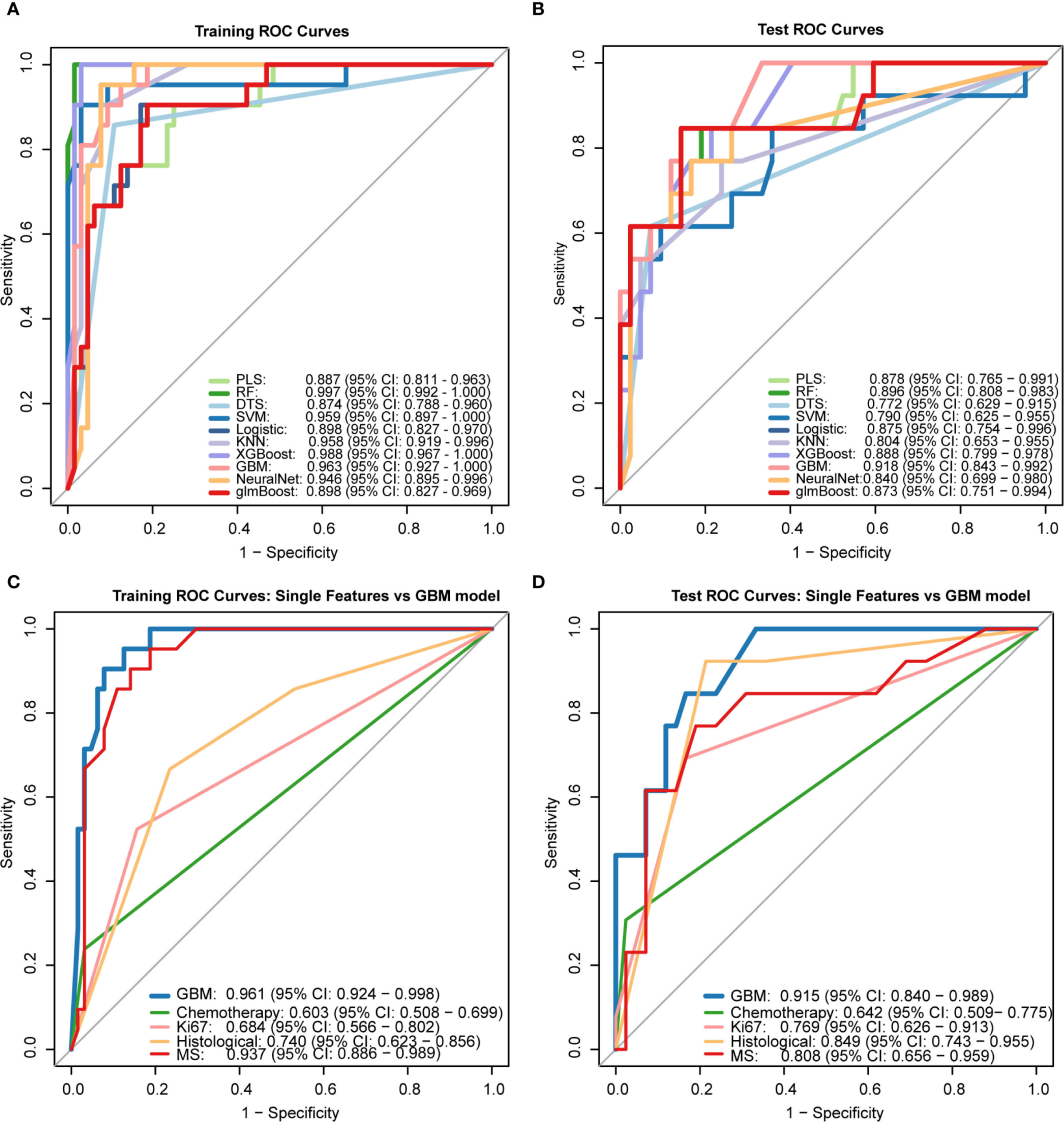
Performance comparison of machine learning models receiver operating characteristic (ROC) curves of 10 ML algorithms in **(A)** training set and **(B)** test set. The Gradient Boosting Machine (GBM) demonstrated superior performance (test AUC = 0.918). **(C, D)** Comparison between GBM integrated model and single-feature signatures, showing higher AUC values for the integrated model in both training (0.961) and test sets (0.915).PLS: Partial Least Squares, RF: Random Forest, DTS: Decision Tree Splitting, SVM: Support Vector Machine, Logistic: Logistic Regression, KNN: K-Nearest Neighbors, XGBoost: eXtreme Gradient Boosting, GBM: Gradient Boosting Machine, NeuralNet: Neural Network, glmBoost: gradient boosting for generalized linear models.

Initially, we hypothesized that a comprehensive integration of clinical features might provide key insights for predicting DCIS recurrence outcomes. Therefore, we obtained 21 clinical features from the electronic medical records and identified four strongly correlated variables through analysis and integration. These four variables were used to develop a predictive model for DCIS recurrence. In the present investigation, ten machine learning algorithms (PLS, RF, DTS, SVM, Logistic, KNN, XGBoost, GBM, NeuralNet, and glmBoost) were evaluated within the discovery dataset to assess their predictive capabilities (**Figures 4A, B**). Based on its superior overall performance across both training and testing datasets, the GBM model was identified as the most effective approach (training set AUC = 0.963, test set AUC = 0.918).Additionally, comparison of the GBM-integrated model with single-risk signatures (**Figures 4C, D**) showed that the GBM-integrated model had the largest area under the ROC curve (AUC) (training set: 0.961; test set: 0.915). Among the single-risk signatures, the MS had the highest AUC in the training set (0.937), whereas histological grading had the highest AUC in the test set (0.849). While individual risk signatures demonstrated measurable net benefit across broad threshold probability ranges, the GBM model exhibited superior overall net benefit performance. Consequently, this model was identified as the most suitable approach for forecasting DCIS recurrence over a five-year period and subsequent timeframes.

**Figure 5:**
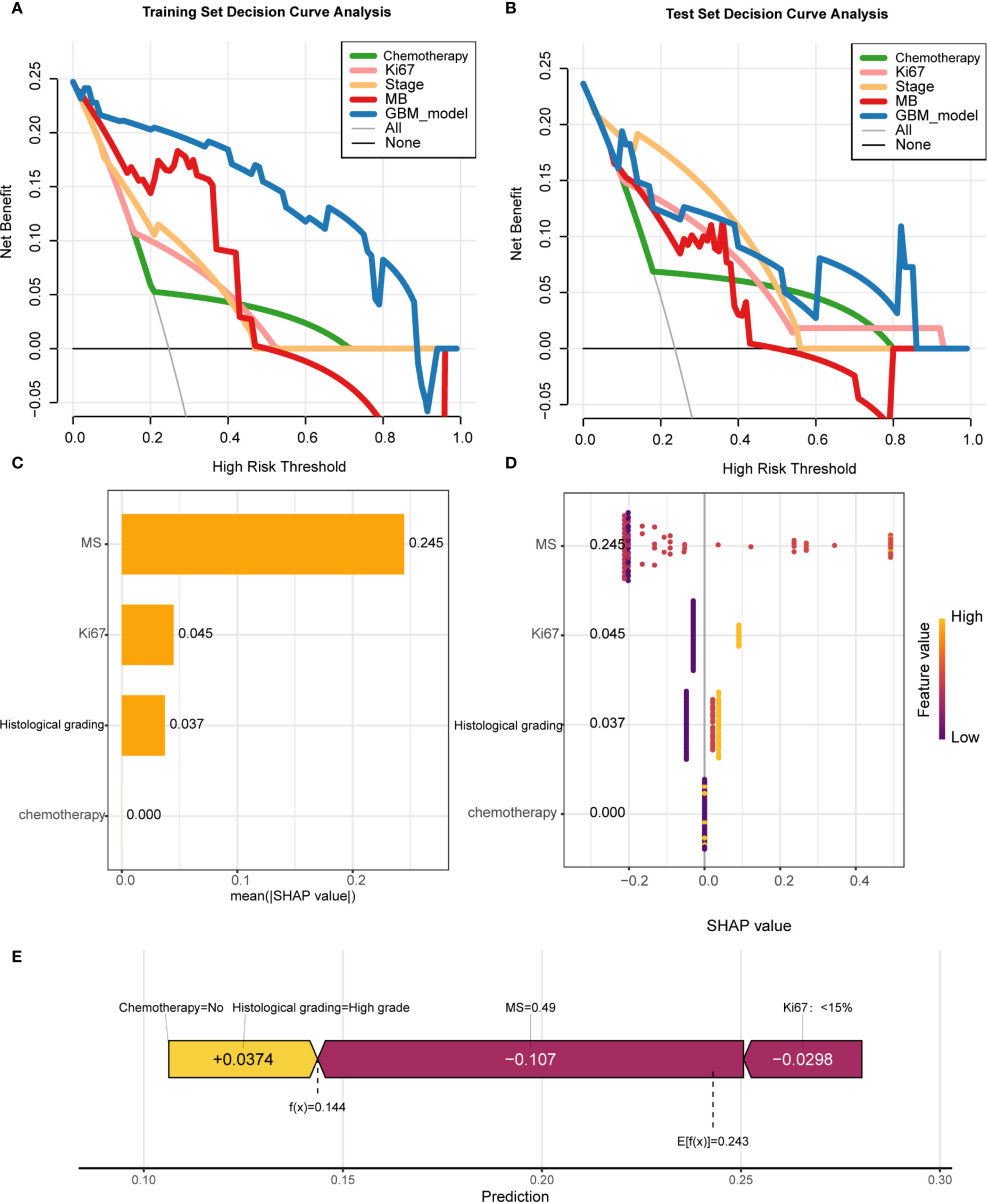
Model interpretability and clinical utility analysis **(A, B)** Decision curve analysis (DCA) demonstrating net benefit of GBM model versus clinical predictors across threshold probabilities. **(C)** SHAP summary plot ranking feature importance (MS > Ki-67 > histological grade > chemotherapy). **(D)** SHAP violin plots showing value distributions impacting predictions (yellow/purple indicate high/low values). **(E)** Force plot exemplifying individualized prediction for a case with reduced recurrence risk (baseline 0.243 → 0.144), where high histological grade was the dominant negative contributor.

Regarding clinical utility, the four-variable model demonstrated substantial net benefits across diverse threshold probability ranges alongside the GBM model; nevertheless, the GBM model displayed superior net benefit performance, thereby validating its designation as the most effective predictive framework for DCIS recurrence (**Figures 5A, B**).To improve model interpretability, we employed the SHAP framework. According to the importance ranking based on the mean absolute SHAP values (**Figure 5C**), the four features were ordered as follows: MS > Ki-67 index > histological grading > chemotherapy status. **Figure 5D** shows a violin plot for each feature, illustrating the correlation between features and SHAP values. Larger absolute SHAP values indicate a greater impact of the features on the GBM-based prediction model. The yellow and purple dots represent higher and lower feature values, respectively. These results underscore that MS was the most critical factor, surpassing Ki-67, histological grade, and chemotherapy. **Figure 5E** presents a comprehensive case analysis illustrating the model’s predictive methodology for an individual patient. Within this representation, yellow markers signify positive influences on the prediction outcome, while purple markers indicate negative influences. The f(x) value corresponds to the computed SHAP value for each contributing factor. Notably, the GBM model predicted a lower recurrence risk than the baseline in this patient. Among these factors, a high histological grade was the primary negative contributor (reducing the predicted risk by -0.107, from a baseline of 0.243 to 0.144), whereas the absence of chemotherapy had a small positive impact (+0.0374). Overall, the combined effects resulted in a prediction that was significantly below the average risk.

## Discussion

This investigation involved the development and performance evaluation of 10 ML algorithms utilizing 21 clinical parameters, encompassing clinical characteristics, mammographic imaging data, and histopathological findings, to forecast long-term recurrence (≥5 years) in DCIS patients following BCS. The findings demonstrated that the GBM model achieved optimal performance with a test set AUC of 0.918, displaying superior predictive capacity compared to the remaining four individual risk signatures and consequently providing substantial clinical utility. To enhance model interpretability, we employed the SHAP methodology for visualization purposes. SHAP force plots were utilized to elucidate the individualized prediction process for DCIS recurrence risk assessment, thereby facilitating comprehensive understanding of the underlying predictive mechanisms (29). While existing clinical risk assessment tools like the VNPI and RTOG 9804 standard rely on traditional clinical-pathological variables (such as age, tumor size, margin width, and histological grade), our approach integrates deep learning-derived breast X-ray imaging features with key clinical predictors. This enables our model to capture tumor heterogeneity and radiologically-based disease progression patterns that conventional scoring systems cannot reveal. Unlike these early-stage tools, our model provides personalized, interpretable risk predictions through SHAP analysis, thereby revealing which factors contribute most significantly to individual recurrence risks.

Most recent studies on predicting recurrence after BCS for DCIS rely solely on single data sources—such as clinicopathological features or imaging indicators—and do not integrate multimodal data, which may lead to the omission of key predictive information (10, 31–33). Second, although existing DL models have certain predictive capabilities (34, 35), they are mostly “black-box” models that lack quantitative explanations of predictive factors, thereby limiting clinician trust in the results. Finally, some studies have established predictive models without sufficient clinical translation validation, lacking both DCA to demonstrate the clinical net benefit and association with specific treatment decisions, thus restricting the model’s practical application. For example, some studies used only molecular phenotypes or pathological information for predictive analysis or performed risk stratification based solely on radiomic features (10, 33); however, these methods have shortcomings in prediction accuracy and clinical applicability.

In the present study, the GBM algorithm, an advanced ensemble learning method based on gradient boosting, demonstrated excellent predictive performance in medical datasets with complex feature interactions. By organically combining DL features from mammography with clinicopathological variables, the GBM model significantly outperformed traditional ML methods in predicting the risk of DCIS recurrence. Compared with traditional LR models, the GBM algorithm more effectively captures nonlinear relationships and feature interactions through its iterative boosting process while maintaining strong robustness to data noise and outliers. SHAP value analysis quantitatively showed that the MS was the most influential predictor, followed by Ki-67 index and histological grade, consistent with the clinical knowledge of DCIS progression. The MS is a composite quantitative score derived from deep learning analysis of preoperative breast X-ray imaging. Although algorithm-generated, its numerical value correlates with visually identifiable radiological features associated with known and invasive lesions. Higher MS scores typically correspond to breast X-ray imaging manifestations characterized by: cluster-like microcalcifications without masses (such as fine speckled, linear, and branched patterns), which may also present as single or multiple masses, particularly those showing a mouse-tail-like blurring at the posterior edge along ductal pathways. The model’s excellent predictive performance (AUC = 0.918) benefits from the ability of the GBM algorithm to process high-dimensional feature spaces while effectively avoiding overfitting through regularization. More importantly, the introduction of SHAP interpretation provides clinicians with transparent model decision-making bases, effectively addressing the common “black box” problem of complex ML models. This optimal combination of prediction accuracy and interpretability renders our GBM framework suitable for decision support in the clinical management of DCIS.

Earlier research has demonstrated that adjuvant radiotherapy substantially diminishes the likelihood of local disease recurrence following breast-conserving surgery for ductal carcinoma *in situ* (hazard ratio 0.3–0.5) (36, 37), findings that align with our current investigation, which revealed a markedly elevated recurrence rate among patients not receiving radiotherapy. However, SHAP analysis in this study further revealed increased recurrence risk even in patients who received radiotherapy, with high-grade lesions, and with high Ki-67 expression, thus suggesting the need to additionally consider molecular characteristics to optimize radiotherapy indications. Our research integrates breast X-ray deep learning features with Ki-67 and histological grading to establish a refined pre-treatment risk stratification framework that surpasses traditional factors. SHAP analysis demonstrates that these elements exhibit additive and potentially synergistic predictive value. For instance, a patient with high-grade DCIS exhibiting high MS (indicating fine linear calcifications or spiculated masses) coupled with elevated Ki-67 levels (>30%) would be identified by our model as having extremely high recurrence risk. This specific imaging-clinical profile suggests a biologically aggressive tumor with high proliferative potential, even in cases of negative margins. While standard adjuvant radiotherapy benefits such high-risk patients, it may not sufficiently counteract their inherent recurrence risk. Consequently, our model serves as a decision-support tool to enhance treatment strategies (including optimized radiotherapy dosage, extended endocrine therapy, consideration of systemic chemotherapy, and intensified monitoring). Conversely, patients with low MS (indicating benign-like features), low histological grade, and low Ki-67 levels are predicted to have excellent prognosis. For these patients, our model supports step-down therapy—such as omitting radiotherapy or following standard monitoring protocols in selected cases—to avoid overtreatment and reduce side effects.

While our model demonstrates strong discriminative capabilities, several critical limitations of this study must be noted. Firstly, the relatively limited sample size—particularly when compared to the original high-dimensional feature set—may raise concerns about overfitting due to the reduced number of events. To address this, we implemented rigorous feature reduction techniques (such as LASSO regression) and cross-validation to mitigate these issues. However, some highly complex models (such as decision trees and extreme random forests) still exhibited overfitting on the training set (AUC = 1.000), highlighting the importance of rigorous model selection and validation in high-dimensional data. The GBM model we ultimately selected demonstrated outstanding and consistent performance across both training and test sets, indicating its strong generalization capability. Nevertheless, this result should be viewed with caution. Secondly, this is a retrospective study conducted by a single institution, with its sample exclusively drawn from the Asian (China) population. The inherent selection bias in this design, combined with the homogeneity of genetic background, lifestyle patterns, and healthcare practices within the population, severely limits the external validity and generalizability of our predictive model. Therefore, before implementing this model in clinical practice, it must undergo large-scale external validation in multicenter prospective cohorts encompassing diverse geographic distributions, ethnicities, and racial backgrounds. Future research should focus on validating the model’s robustness through larger-scale samples. Only after demonstrating its effectiveness across broader populations can this model be considered a universally applicable decision support tool. Thirdly, regarding post-breast-conserving surgery adjuvant therapy details, our study is constrained by the completeness of available retrospective data. While we documented the implementation of adjuvant chemotherapy, endocrine therapy, and adjuvant radiotherapy, specific protocol details (such as chemotherapy cycle counts and dosages; endocrine drug selection and treatment duration; total radiation dose, fractionated regimens, and brachytherapy usage) and technical specifications were not consistently available for all patients. Consequently, our analysis could not account for potential variations in radiotherapy protocols that might influence recurrence outcomes and constitute unmeasured sources of confounding factors. Finally, while multimodal data were integrated, the extraction of imaging features mainly relied on two-dimensional mammography images and did not include richer imaging information such as dynamic contrast-enhanced MRI.

## Conclusion

This research documented the utilization of machine learning methodologies incorporating mammographic imaging characteristics, clinical data, and laboratory measurements for forecasting recurrence among DCIS patients, establishing a GBM algorithmic framework capable of accurately estimating DCIS recurrence probability. In this study, the combination of ML with the interpretable SHAP method endowed the “black-box” ML model with interpretability, making it more suitable for predicting DCIS recurrence in clinical scenarios. Additionally, the inclusion of DCA highlights the clinical value of GBM. We propose the use of this approach as an auditable decision-support tool to facilitate patient healthcare and research.

## Data Availability

The original contributions presented in the study are included in the article/**Supplementary Material**. Further inquiries can be directed to the corresponding author/s.

## References

[1] Kim J Harper A McCormack V Sung H Houssami N Morgan E. Global patterns and trends in breast cancer incidence and mortality across 185 countries. Nat Med. (2025) 31:1154–62. doi: 10.1038/s41591-025-03502-3, PMID: 39994475

[2] Farante G Toesca A Magnoni F Lissidini G Vila J Mastropasqua M. Advances and controversies in management of breast ductal carcinoma in *situ* (DCIS). Eur J Surg Oncol. (2022) 48:736–41. doi: 10.1016/j.ejso.2021.10.030, PMID: 34772587

[3] Wärnberg F Garmo H Emdin S Hedberg V Adwall L Sandelin K. Effect of radiotherapy after breast-Conserving surgery for ductal carcinoma in situ: 20 years follow-Up in the randomized sweDCIS trial. J Clin Oncol. (2014) 32:3613–8. doi: 10.1200/JCO.2014.56.2595, PMID: 25311220

[4] Maxwell AJ Hilton B Clements K Dodwell D Dulson-Cox J Kearins O. Unresected screen-detected ductal carcinoma in *situ*: Outcomes of 311 women in the Forget-Me-Not 2 study. Breast. (2022) 61:145–55. doi: 10.1016/j.breast.2022.01.001, PMID: 34999428 PMC8753270

[5] Teh Y-C Tan G-H Taib NA Rahmat K Westerhout CJ Fadzli F. Opportunistic mammography screening provides effective detection rates in a limited resource healthcare system. BMC Cancer. (2015) 15:405. doi: 10.1186/s12885-015-1419-2, PMID: 25972043 PMC4437679

[6] Dershaw DD Abramson A Kinne DW. Ductal carcinoma in *situ*: mammographic findings and clinical implications. Radiology. (1989) 170:411–5. doi: 10.1148/radiology.170.2.2536185, PMID: 2536185

[7] Grimm LJ Rahbar H Abdelmalak M Hall AH Ryser MD. Ductal carcinoma in situ: state-of-the-art review. Radiology. (2022) 302:246–55. doi: 10.1148/radiol.211839, PMID: 34931856 PMC8805655

[8] Tan H Wu Q Wu Y Zheng B Wang B Chen Y. Mammography-based artificial intelligence for breast cancer detection, diagnosis, and BI-RADS categorization using multi-view and multi-level convolutional neural networks. Insights into Imaging. (2025) 16:109. doi: 10.1186/s13244-025-01983-x, PMID: 40397242 PMC12095762

[9] Khalid A Mehmood A Alabrah A Alkhamees BF Amin F AlSalman H. Breast cancer detection and prevention using machine learning. Diagnostics. (2023) 13:3113. doi: 10.3390/diagnostics13193113, PMID: 37835856 PMC10572157

[10] Alaeikhanehshir S Voets MM van Duijnhoven FH lips EH Groen EJ van Oirsouw MCJ. Application of deep learning on mammographies to discriminate between low and high-risk DCIS for patient participation in active surveillance trials. Cancer Imaging. (2024) 24:48. doi: 10.1186/s40644-024-00691-x, PMID: 38576031 PMC10996224

[11] Ponce-Bobadilla AV Schmitt V Maier CS Mensing S Stodtmann S. Practical guide to SHAP analysis: Explaining supervised machine learning model predictions in drug development. Clin Trans Sci. (2024) 17:e70056. doi: 10.1111/cts.70056, PMID: 39463176 PMC11513550

[12] Guan X Du Y Ma R Teng N Ou S Zhao H. Construction of the XGBoost model for early lung cancer prediction based on metabolic indices. BMC Med Inf Decision Making. (2023) 23:107. doi: 10.1186/s12911-023-02171-x, PMID: 37312179 PMC10262551

[13] Yogananda CGB Shah BR Vejdani-Jahromi M Nalawade SS Murugesan GK Yu FF. A fully automated deep learning network for brain tumor segmentation. Tomography. (2020) 6:186–93. doi: 10.18383/j.tom.2019.00026, PMID: 32548295 PMC7289260

[14] Broc C Truong T Liquet B. Penalized partial least squares for pleiotropy. BMC Bioinf. (2021) 22:86. doi: 10.1186/s12859-021-03968-1, PMID: 33627076 PMC7905667

[15] Chen Z He N Huang Y Qin WT Liu X Li L. Integration of A deep learning classifier with A random forest approach for predicting malonylation sites. Genomics Proteomics Bioinf. (2019) 16:451–9. doi: 10.1016/j.gpb.2018.08.004, PMID: 30639696 PMC6411950

[16] Yang Y Xu L Sun L Zhang P Farid SS. Machine learning application in personalised lung cancer recurrence and survivability prediction. Comput Struct Biotechnol J. (2022) 20:1811–20. doi: 10.1016/j.csbj.2022.03.035, PMID: 35521553 PMC9043969

[17] Vetter TR Schober P. Regression: the apple does not fall far from the tree. Anesth Analgesia. (2018) 127:277–83. doi: 10.1213/ANE.0000000000003424, PMID: 29771712

[18] Bian Z Vong CM Wong PK Wang S. Fuzzy KNN method with adaptive nearest neighbors. IEEE Trans Cybernetics. (2022) 52:5380–93. doi: 10.1109/TCYB.2020.3031610, PMID: 33232252

[19] Ma B Meng F Yan G Yan H Chai B Song F. Diagnostic classification of cancers using extreme gradient boosting algorithm and multi-omics data. Comput Biol Med. (2020) 121:103761. doi: 10.1016/j.compbiomed.2020.103761, PMID: 32339094

[20] Rodríguez-Tomàs E Arenas M Baiges-Gaya G Acosta J Araguas P Malave B. Gradient boosting machine identified predictive variables for breast cancer patients pre- and post-Radiotherapy: preliminary results of an 8-Year follow-Up study. Antioxidants. (2022) 11:2394. doi: 10.3390/antiox11122394, PMID: 36552602 PMC9774765

[21] Zhao S Chen P Wang X Zheng Z Hui R Pang G. Preoperatively predicting human epidermal growth factor receptor 2-low expression in breast cancer using neural network model based on multiparameter magnetic resonance imaging. Quantitative Imaging Med Surg. (2024) 14:8387–401. doi: 10.21037/qims-24-428, PMID: 39698610 PMC11652052

[22] Wu Y Xu D Zha Z Gu L Chen J Fang J. Integrating radiomics into predictive models for low nuclear grade DCIS using machine learning. Sci Rep. (2025) 15:7505. doi: 10.1038/s41598-025-92080-y, PMID: 40033061 PMC11876686

[23] Ruz GA Araya-Díaz P Henríquez PA. Facial biotype classification for orthodontic treatment planning using an alternative learning algorithm for tree augmented Naive Bayes. BMC Med Inform Decis Mak. (2022) 22:316. doi: 10.1186/s12911-022-02062-7, PMID: 36456974 PMC9713997

[24] Liang Y Ma X. iACP-GE: accurate identification of anticancer peptides by using gradient boosting decision tree and extra tree. SAR QSAR Environ Res. (2023) 34:1–19. doi: 10.1080/1062936X.2022.2160011, PMID: 36562289

[25] Wang LA Kern R Yu E Choi S Pan JQ. IntelliSleepScorer, a software package with a graphic user interface for automated sleep stage scoring in mice based on a light gradient boosting machine algorithm. Sci Rep. (2023) 13:4275. doi: 10.1038/s41598-023-31288-2, PMID: 36922536 PMC10017698

[26] Sorayaie Azar A Babaei Rikan S Naemi A Bagherzadeh Mohasefi J Pirnejad H Bagherzadeh Mohasefi M. Application of machine learning techniques for predicting survival in ovarian cancer. BMC Med Inform Decis Mak. (2022) 22:345. doi: 10.1186/s12911-022-02087-y, PMID: 36585641 PMC9801354

[27] Jayaram N Muralidharan M Muthupandian S. The use of multilayer perceptron and radial basis function: an artificial intelligence model to predict progression of oral cancer. Int J Surg. (2023) 109:57–9. doi: 10.1097/JS9.0000000000000026, PMID: 36799795 PMC10389180

[28] Lan X Wang X Qi J Chen H Zeng X Shi J. Application of machine learning with multiparametric dual-energy computed tomography of the breast to differentiate between benign and Malignant lesions. Quantitative Imaging Med Surg. (2021) 12:810–22. doi: 10.21037/qims-21-39, PMID: 34993120 PMC8666765

[29] Miranda E Adiarto S Bhatti FM Zakiyyah AY Aryuni M Bernando C. Understanding arteriosclerotic heart disease patients using electronic health records: A machine learning and shapley additive exPlanations approach. Healthc Inform Res. (2023) 29:228–38. doi: 10.4258/hir.2023.29.3.228, PMID: 37591678 PMC10440196

[30] Wang G Zhang Y Li S Zhang J Jiang D Li X. A machine learning-based prediction model for cardiovascular risk in women with preeclampsia. Front Cardiovasc Med. (2021) 8. doi: 10.3389/fcvm.2021.736491, PMID: 34778400 PMC8578855

[31] Rakovitch E Nofech-Mozes S Hanna W Baehner FL Saskin R Butler SM. A population-based validation study of the DCIS Score predicting recurrence risk in individuals treated by breast-conserving surgery alone. Breast Cancer Res Treat. (2015) 152:389–98. doi: 10.1007/s10549-015-3464-6, PMID: 26119102 PMC4491104

[32] Liu C Sun M Arefan D Zuley M Sumkin J Wu S. Deep learning of mammogram images to reduce unnecessary breast biopsies: a preliminary study. Breast Cancer Res. (2024) 26:82. doi: 10.1186/s13058-024-01830-9, PMID: 38790005 PMC11127450

[33] Wetstein SC Stathonikos N Pluim JPW Heng YJ ter Hoeve ND Vreuls CPH. Deep learning-based grading of ductal carcinoma in *situ* in breast histopathology images. Lab Invest. (2021) 101:525–33. doi: 10.1038/s41374-021-00540-6, PMID: 33608619 PMC7985025

[34] Dong J Feng T Thapa-Chhetry B Cho BG Shum T Inwald DP. Machine learning model for early prediction of acute kidney injury (AKI) in pediatric critical care. Crit Care. (2021) 25:288. doi: 10.1186/s13054-021-03724-0, PMID: 34376222 PMC8353807

[35] Heo J Yoon JG Park H Kim YD Nam HS Heo JH. Machine learning–based model for prediction of outcomes in acute stroke. Stroke. (2019) 50:1263–5. doi: 10.1161/STROKEAHA.118.024293, PMID: 30890116

[36] Corradini S Pazos M Schönecker S Reitz D Niyazi M Ganswindt U. Role of postoperative radiotherapy in reducing ipsilateral recurrence in DCIS: an observational study of 1048 cases. Radiat Oncol. (2018) 13:25. doi: 10.1186/s13014-018-0964-7, PMID: 29426355 PMC5807793

[37] van Seijen M Lips EH Thompson AM Nik-Zainal S Futreal A Hwang ES. Ductal carcinoma in *situ*: to treat or not to treat, that is the question. Br J Cancer. (2019) 121:285–92. doi: 10.1038/s41416-019-0478-6, PMID: 31285590 PMC6697179

