# Supplementary material for: Integrating deep learning features from mammography with SHAP values for a machine learning model predicting over 5-year recurrence of breast ductal carcinoma In Situ post-lumpectomy
Source: Front Immunol. 2025 Sep 15;16:1681072. doi: 10.3389/fimmu.2025.1681072 (PMC12477132; doi:10.3389/fimmu.2025.1681072)
Supplement: Supplementary file 2 [file DataSheet2.pdf]

伦理审查意见

审查日期	2024 年 11 月 28 日	伦理编号	YD2024-18
审查类别	研导项目审查	审查方式	快速审查
审查地点	哈尔滨医科大学附属肿瘤医院		
项目名称	基于 MRI 和病理图像的深度学习模型预测乳腺癌新辅助治疗患者的预后		
方案编号		项目来源	哈尔滨医科大学附属肿瘤医院牛明
主要研究者	牛明	研究科室	乳腺整形外科病房
同意文件	1. 研究方案：版本 1.0/2024-7-21<br>2. 免除知情同意申请：2024-7-21<br>3. 知情同意书：2024-7-21<br>4. 数据记录表：版本 1.0/2024-7-21（回顾、前瞻性研究）		
审查意见	本伦理委员会组成、职责及工作程序严格遵循国际伦理准则、GCP 规范及中国相关的法律法规。伦理委员会审阅了该项目文件（审查文件目录详见附件），评审结果“同意”。根据快审委员审查意见，伦理委员会同意该项目自审批之日起可在本中心开展该项研究，同意免除部分知情同意。要求所有资料未经伦理委员会批准，不得做任何修改。<br><br>主任或副主任委员签字：<br>哈尔滨医科大学附属肿瘤医院伦理委员会（盖章）<br>日期：2024 年 12 月 2 日		
年度/定期跟踪审查频率	12 个月		

备注：（请仔细阅读）

1. 本批件将在立项部门及研究科室备案，批件有效期为1年（解释有效期：本项临床试验应当在批准之日起 1 年内实施。逾期未实施，本批件自行废止）。
2. 若研究获取到中国人类遗传资源管理办公室审批决定书，须在研究启动前递交申请书及批件备案。
3. 对已审查同意的项目须遵循本伦理委员会同意的方案执行，研究须符合《赫尔辛基宣言》、GCP 等要求。
4. 对已审查同意的临床研究方案、知情同意书、招募材料、提供给受试者其他书面材料、病例报告表等的修改或更换主要研究者，须及时向本伦理委员会申请，经审查同意后实施。
5. 根据伦理委员会对年度/定期跟踪审查频率的意见，请在审查日到期前1个月提交研究进展报告。
6. 本中心发生的可疑且非预期严重不良反应/严重不良事件及影响研究风险受益比的安全性信息，须及时报告。
7. 本中心发现重大违背方案情况，须及时向伦理委员会提交违背方案报告。
8. 本中心暂停/终止临床研究，须向伦理委员会提交暂停/终止研究报告。

9. 本中心完成临床研究，须在关中心前，提交研究完成报告，供伦理委员会审查。

附件：伦理委员会审查文件目录

1. 本院涉及人的研究课题申请表（适用于研究生导师）：2024-10-28
2. 科研项目伦理审查申请表
3. 学位论文开题报告及课题计划：2024-7-21
4. 研究方案：版本 1.0/2024-7-21
5. 免除知情同意申请：2024-7-21
6. 哈尔滨医科大学附属肿瘤医院《病史数据/生物标本二次利用知情同意书》（适用于申请免知情）医疗表格：编号 5-37
7. 知情同意书：1.0/2024-7-21
8. 课题团队名单
9. 课题负责人简历、GCP 证书复印件
10. 数据记录表：版本 1.0/2024-7-21（回顾、前瞻性研究）
11. 研究者导师课题伦理批准后开展承诺书：2024-10-28

哈尔滨医科大学附属肿瘤医院第三届伦理委员会名单

委员会职务	姓　名	性别	专业背景	职　称	工作单位
主任委员	郑桐森	男	临床医学	主任医师	哈医大附属肿瘤医院
副主任委员	张艳桥	女	临床医学	主任医师	哈医大附属肿瘤医院
委员	赵长宏	男	临床医学	主任医师	哈医大附属肿瘤医院
委员	冯小东	男	药学	副主任药师	哈医大附属肿瘤医院
委员	蔡　莉	女	临床医学	主任医师	哈医大附属肿瘤医院
委员	于　雁	女	临床医学	主任医师	哈医大附属肿瘤医院
委员	李玉莲	女	临床医学	主任医师	哈医大附属肿瘤医院
委员	苏　君	女	临床医学	主任医师	哈医大附属肿瘤医院
委员	孙文洲	男	临床医学	主任医师	哈医大附属肿瘤医院
委员	李志伟	男	临床医学	主任医师	哈医大附属肿瘤医院
委员	柳　萍	女	管理	研究员	哈医大附属肿瘤医院
委员	陈　旭	女	法学	律　师	黑龙江大公律师事务所
委员	董　梅	女	药学	主任药师	哈医大附属肿瘤医院
委员	周桂敏	女	工业锅炉	工程师	社会退休人士
委员	马　荣	女	肿瘤学	主任医师	哈医大附属肿瘤医院
委员	刘瑞宝	男	影像医学与核医学	主任医师	哈医大附属肿瘤医院
委员	张显玉	男	肿瘤学	研究员	哈医大附属肿瘤医院
委员	袁贵生	男	社会医学与卫生事业管理	研究员	哈医大附属肿瘤医院
委员	尹　梅	女	医学伦理学	教　授	哈尔滨医科大学
委员	孟宏学	男	病理学	主任医师	哈医大附属肿瘤医院
委员	尹　航	男	肿瘤学	副主任医师	哈医大附属肿瘤医院
委员	王子琦	男	外科学	副主任医师	哈医大附属肿瘤医院

声明：

1. 本伦理委员会组成、职责及工作程序严格遵循国际伦理准则/指南、GCP 规范及中国相关的法律法规。

2. 此名单为哈尔滨医科大学附属肿瘤医院第三届伦理委员会换届后第五次调整形成，所有委员均在有效任职期内。
